# Art of publication: A beginner’s guide to understanding the non-linear dynamics between research and publication

**Published:** 2021-06-30

**Authors:** BIPLAB SARKAR

**Affiliations:** Chief Medical Physicist, Department of Radiation Oncology Apollo Multispeciality Hospitals, Kolkata, West Bengal, India

## INTRODUCTION

Unlike the mainstream academic curriculum, research orientation is not part of the hospital-based medicine syllabus. At present, there is a dearth of training in research for young scholars starting their careers as junior residents for obtaining a postgraduate degree in any discipline of medicine. These individuals often experience helplessness as untrained researchers. It is evident that the current academic system, especially in developing countries, is not going to evolve in the near future or offer appropriate training in research methodology and publication. Therefore, the last resort is to go through a self-learning module on how to conduct research and publish articles. This article is designed as a self-help module for those interested in research and publication, particularly in the field of oncology.

Why do researchers publish articles? The most appropriate reason would be that they have discovered some new science/philosophy and would like the world to know about it. A less altruistic and rather materialistic reason is to get higher academic degrees, such as Doctor of Philosophy (PhD) and Doctor of Medicine (MD).

For a young researcher who aspires to have a good publication record, it is indispensable to know the methodology of publishing an article. Discovering new science although a prerequisite may not be sufficient for the publication of an article. The hurdles associated with a good publication include a flawed study design and inappropriate methodology, lack of clarity and reproducibility, and most importantly, difficulty in translating research into a publication.^[1]^ Therefore, the art of publishing is not proportional to the significance of the invention; the dynamics are typically non-linear.

A search on Google Scholar with the search strings, “how to write a scientific paper” and “how to write a paper” returned about 1.8 million and 3.7 million results, respectively, making it one of the most investigated topics. There is plenty of literature in the form of articles and books which describes the technique of writing a paper. Numerous authors have already elaborately discussed how to write scientific articles and respond to critics.^[1,2]^ Therefore, in this article, I have attempted to briefly discuss only the salient features of the technique of writing a research paper.

**Figure 1:**
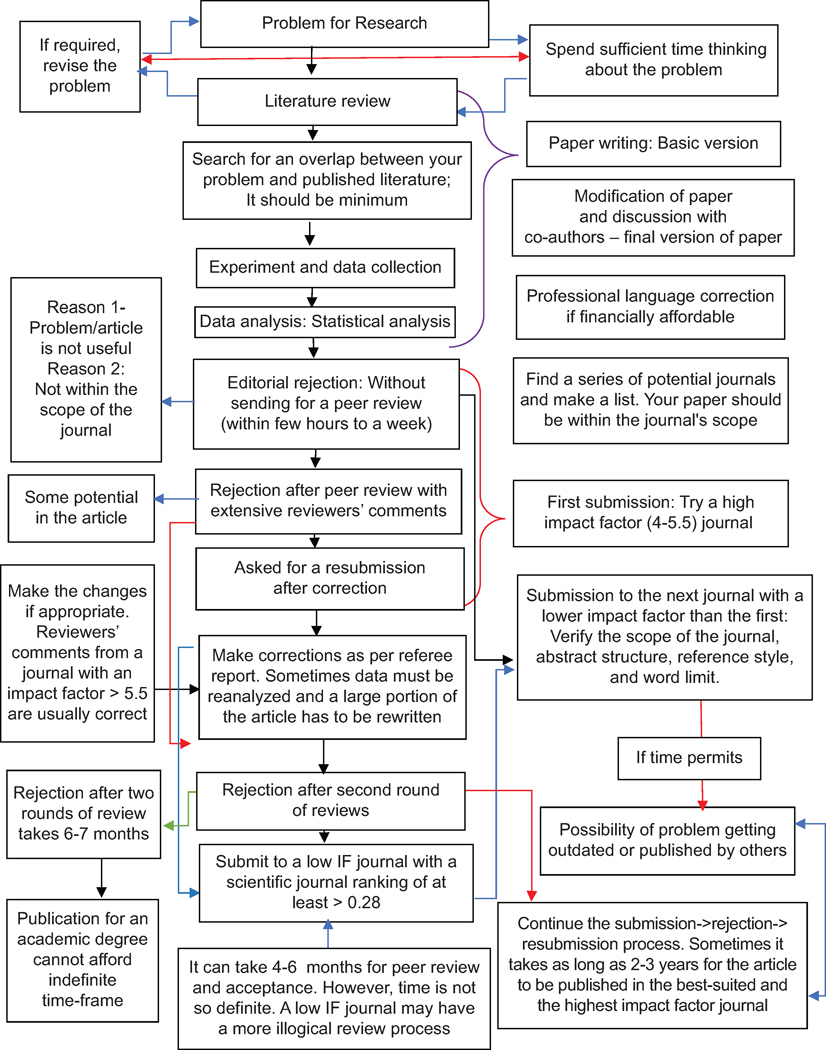
Publication flowchart

This article provides guidelines for research beginners on how to publish scientific articles, with a detailed flowchart of the various stages of publication [Figure 1]. Although these guidelines can be applied across all the disciplines of science, technology, and medicine, they have been described in the context of oncology and its associated interdisciplinary subjects, like medical physics. Additionally, the issues faced during publication by those pursuing higher academic degrees such as MD, Master of Surgery (MS), Diplomate of National Board (DNB), and PhD in India are also discussed.

## CHALLENGES

Cancer research is one of the most globally active domains of science. Between 2001 and 2015, a total of 62,550 research articles on radiotherapy were published from 127 countries in 2531 international journals.^[3]^ The difficulties associated with publishing in oncology in developing and at times even in developed countries, include a lack of systematic research programs, except in a few institutions, reluctance and ignorance about publishing, lack of dedicated time for research amidst clinical, academic, regulatory, and administrative work, no academic or financial credit for research and publication as the clinical establishments are not under the direct supervision of any university, lack of training in how to publish, very poor scientific language (namely English), lack of guidance from a supervisor (PhD guide or co-guide), lack of a sustainable attitude to make repeated corrections in an article, dearth of instruments, resources or facilities, and absence of access to journals/literature.

## HOW CAN ONE OVERCOME THE CHALLENGES?

### Practice of thinking

The most important point for research, and hence for publication, is the habit of constructive and sustained thinking about a problem. This, in turn, builds an attitude to tackle any given problem in a scientific manner.

The technique of publication encompasses cultivating and fostering a new idea. Whatever be the idea, one should spend considerable time thinking and working on it. Although not essential, it could be useful to get help from others.

### Time management

Medical professionals need to find time regardless of their busy schedules. However, sometimes, people keep themselves busy in unproductive work; therefore, individuals or groups need to rationalize how they spend their time and find suitable intervals for research work. Time management comes from the consciousness, urge, and dedication towards research and publication. With smart and parallel workflow management, it is possible to spare about 3–3.5 hours per day for research and publication. Writing 5–10 lines a day is more than sufficient for publishing more than four articles in a year. However, writing five lines a day is possible only when someone has been thinking about the problem for hours or days.

### Lack of equipment and facilities

Lack of machines or facilities is a hostile situation for researchers in developing and underdeveloped countries.^[4]^ However, researchers should not get overwhelmed in such situations as they hold very little significance for publication. The research design should be aligned with the available facilities. An advantage of a setup that lacks facilities is the availability of a large number of patients, which can be thoughtfully exploited for research. Oncology is a field where the most important aspects of research are patient follow-up, survival, fear psychosis, mental agony, and financial distress. These subjects are extremely vast and selectively ignored in the present context of oncology research in developing countries. In the field of oncology and medical physics, patients are more important than the technology.

### Lack of access to literature

Not having access to literature is a serious problem and can be circumvented partially by using various networking sites and search engines such as ResearchGate and Google Scholar.^[5]^ Articles can be requested from the corresponding author(s), and usually, they fulfill the request. Several countries have World Health Organization-sponsored Hinari/Research for health initiatives, a program under which all the literature is freely available.^[6]^

### Literature review

This is the most important aspect of publishing a research article. Incorrect or insufficient literature review may lead to working on an already solved problem, both for a standalone experiment or an MD/PhD thesis. PubMed and Google Scholar can be used effectively for a literature search. For example, if a researcher wants to work on electronic portal imaging device (EPID) dosimetry, or more specifically forward-and back-projection EPID dosimetry, he/she should use the search term “EPID Dosimetry” in Google Scholar. The search will return a list of articles sorted according to their relevance with the most cited ones being displayed first. Furthermore, filters can be used to display only the recently published articles. For instance, I restricted my search to the articles published in 2018 and found the article titled, “Comparison of forward-and back-projection *in vivo* EPID dosimetry for volumetric-modulated arc therapy (VMAT) treatment of the prostate” published in *Physics in Medicine and Biology*.^[7]^

The article is referenced comprehensively, and reviewing these references partially or completely can provide insights into the work carried out in forward- and back-projection EPID dosimetry so far.

Citing sources and building bibliographies is a complex process. It gets even more complex if researchers are at the end of a big project like PhD and begin to organize and cite the references. One can use a reference management tool like EndNote for organizing the references.

### Methods/experiment and data collection

It is essential to realize how an experimental outcome will be beneficial for society. The likelihood of publication of an article is directly proportional to its applicability in the society. Therefore, it is necessary to preserve the experimental setup, if possible, until the data are published; sometimes, based on the reviewers’ comments, researchers might have to perform new experiments or reanalyze the data. In studies involving human subjects (like clinical outcome evaluation or toxicity measurement), it is essential to get approval from an institutional ethics committee/institutional review board along with registering the project in a publicly accessible clinical trials registry.^[8]^

### Structural and linguistic correction

This is divided into two parts, (i) writing and structuring of the article, i.e., editorial corrections and (ii) linguistic corrections; these broadly include the corrections pertaining to the use of scientific English. Both of these areas are equally important. Following are some important guidelines on writing and structuring the article. Create a basic outline of the salient features of the study. Structure the article in the IMRAD format IMRAD format (Introduction + Methods + Results + Discussion and Conclusion sections).^[9]^ Write down the important points and arguments that should be discussed in each section. An elaborate discussion on paper writing skills can be found in Grech *et al*. and Maloy’s articles.^[1,2]^

It is essential for untrained researchers to express their ideas in proper scientific English. Articles not written in scientific English often face rejection. Emphasis on grammatical and linguistic correctness by reviewers is justified from the perspective of the readers’ understanding of the article. A reviewer may not always be ready to overlook the poor linguistic quality of an article. However, if the scientific content of the article is of high quality, the reviewer might suggest professional language correction. Publication-grade English language correction can be performed within 3–5 days by professional language editors. However, such services often do not include an editorial or structural correction. Scientific English editing is a flourishing business, and these services are offered by all the leading publishers. Universities also offer grammatical correction services at nominal rates to their research scholars. If the topic of research is good, it is advisable to seek professional language correction as this significantly increases the chance of acceptance for publication in major journals.

Following is the unconventional way of making linguistic and structural corrections to a research article. Select one or two articles published in high-impact journals. These articles should be relevant to your area of research. Mimic the structural and preferably the linguistic flow of the article. This will help produce the first draft of the manuscript, which can be further modified especially in terms of the scientific content. However, it is essential to note that it is not advisable to blindly copy the contents from published literature as this would constitute plagiarism. One may seek help from an amateur language editor from the scientific community. A few senior editors may also be willing to perform language corrections; they can be rewarded by offering them co-authorship on the article.

### Rejection, publication time frame, and record-keeping

Article rejection is the biggest reality in the life of any researcher. Even though it can be frustrating, it should be taken in stride. The peer-review process at times is very subjective and may not be able to evaluate the actual potential of the article. However, currently, we do not have any better methods than the present peer-review system. There are different kinds of peer-review processes, discussion on these processes is beyond the scope of this article; however, it is advisable to consider journals that follow a double-blinded peer review, as their review process is free from any kind of bias and provides a fair judgment on the article. For example, the reviews from the *International Journal of Radiation Oncology Biology and Physics* and for *Cancer Research, Statistics*, and *Treatment* are always very fair as they follow a double-blinded peer-review policy.

The time frame for a paper to get published may vary from 6 months (one or two journals) to as high as 3 years (≥10 journals). This can be attributed to several factors, the most prominent ones being the scientific content of the article and the scope of the journal. I wrote two articles that recently got published: the first one got published after 5 months, whereas the second one took 2.8 years.^[10,11]^

In case of delayed publication, meticulous record-keeping is essential. Often, the referee questions are repeated between journals during various rounds of revisions. These repeated questions can be easily answered if there is an organized record of the comments from the previous referees and their responses. A PhD scholar cannot afford an indefinite timeline for publication; therefore, it is necessary to consider the optimal publication time frame when choosing potential journals.

### Selection of MD/MS/PhD thesis topic

Selection of a topic for a degree can be tricky, but it is often supervised by the guide or co-guide. In case of lack of guidance for the selection of the topic, one must choose a broad area of research. For an MD degree in any of the oncology disciplines, the topic should be up to date and should not be a repetition of previous work. Topics such as tumor recurrence and survival analysis are interesting; however, these are often ignored as most students prefer to study the difference in the efficacy between two drugs or aspects related to surgical and radiation techniques. Only a few publish their MD thesis work. This is a drawback of the system which does not encourage its students to explore other areas of research.

In several developing countries, students in the discipline of Medical Physics receive their PhD degrees in Physics or allied subjects instead of Medical Physics because of the unavailability of a dedicated department for this subject in the universities. However, scholars should try to keep the study restricted to medical physics, radiation oncology, biomedical engineering, or computational techniques.

Researchers who are good at mathematics should make an effort to blend medical physics with mathematics. Relevant mathematical formulation can help to get a paper published easily. PhD scholars who lack guidance for choosing a thesis topic should also select a broad and accommodative topic of research. This may help in getting a number of related publications required for the PhD thesis.

### Transformative, open access, and predatory journals

In general, universities accept the publications in the journals included in the Scopus list of journals, and in the science citation index (SCI) list.^[12–14]^ Publications not related to attainment of one’s degree should preferably be in SCI/Scopus journals. Scopus contains 37,535 journals across all subjects. Specifically for publishing in oncology journals, a master list is available from “Cancer Index;” this includes journals from all oncology specialties.^[15,16]^ However, for Medical Physics, such a list of journals is not available. A summarized list of journals for oncology and medical physics is provided in Table 1, along with their scientific journal rankings (SJRs).

Journals can be categorized as (1) open accesses (OA) – without article processing charges (APCs), (2) subscription based-transformative journals, or (3) OA with APC. Few universities discourage publishing in OA with APC journals for MD/PhD degrees. Additionally, as APCs are high, research groups without financial support should avoid such journals.^[17]^

One should be careful not to have one’s articles published in a predatory journal(s). A predatory journal has no peer-review system or editorial board, and often seeks articles from the authors through personal communication. These journals publish worthless articles and sometimes ask for APC after claiming that the article has been accepted for publication.

The best way to avoid predatory journals is to select a target journal for publication from the Scopus database and to consider the SJR. Journals with an SJR and those listed in Scopus are not predatory journals.

## DISCUSSION

### Common issues when choosing a research topic

Repetition of research that has already been done is the most common issue in an unsupervised research project conducted by an untrained researcher. The three typical examples of a research topic for each modality of oncology practice are (1) efficacy of concurrent chemotherapy (temozolomide) and radiotherapy in glioblastoma, (2) describing the surgical technique of radical neck dissection for head-and-neck cancer, and (3) dosimetric comparison between VMAT and intensity-modulated radiotherapy for prostate cancer. As these topics are well researched, the findings cannot be published unless they are new. The choice of topic should therefore include something new and its finite yield.

### Limited and extended research career

It is important to understand that not everyone will publish many articles. In the western and developed countries as well, only a fraction of the total number of practicing oncologists/medical physicists are engaged in research. For medical physicists not working in academic institutions, it is not essential to have a PhD degree; however, it is strongly recommended. A PhD degree can uplift an individual to the level of his/her counterpart in other fraternities like teaching (assistant professor) or medicine (MD/DNB). In such cases, a limited research career of 5–7 years can be opted for.

Contrarily, people who are passionate about research should opt for an extended research career and things will automatically evolve for the individual over time; no specific suggestion is required.

### Impact factor and its significance

This is a term known to everyone, but the concept can be rather confusing.^[18]^ Mushrooming journals add to this confusion by claiming high impact factors (IFs) for themselves. Often, journals claim IFs and listings such as the global impact factor (GIF), universal impact factor (UIF), Citefactor, KoreaMed, Synapse, PubMed, PubMed Central, and Google Scholar. GIF, UIF, and Citefactor are fake IFs and damage the prestige and reliability of scientific research and scholarly journals.^[19]^ The other websites such as KoreaMed, Synapse, PubMed, PubMed Central, and Google Scholar work more like search engines. The only dependable IF is the Thomson Reuters (IF) through the SCI or extended SCI (eSCI). This article exclusively reports the Thomson Reuters IFs available from Clarivate analytics.^[20]^

The IFs of oncology journals vary widely, with the highest among all journals across all disciplines being 292.27 in 2019 for CA – *Cancer Journal for clinicians*. In general, any oncology journal with an IF between 3.0 and 6.5 is considered good for publication.^[12]^ However, for beginners, it may be challenging to publish an article in a journal with an IF of 2.5; therefore, they can consider journals with an IF between 0.7 and 2.5. A series of recommended journals in medical physics is listed in Table 1. Beginners may consider journals with zero IF for publication. However, it must be ensured that the journal has an SJR preferably of over 0.2. SJR is a more stable parameter than IF for measuring the consistency of a journal’s performance.

### Present scenario and future perspectives

Young professionals with a research inclination often ask, “I read and understood an article; what should I do next?” The answer is to think it over for a sufficient period of time. Although this is a philosophically correct answer, it may not convey the intended message to the person asking this question. Therefore, I decided to write this article after one and a half decade of extensive experience in research and publication. It is essential to start from scratch by looking at each problem from the first principle. Individuals who develop the ability to think without any help or guidance from others and gain considerable experience in research often come up with many innovative world class ideas.

## CONCLUSION

Publishing is a skill primarily based on the sustained practice of thinking, which needs to be acquired over the years with or without any guidance. Oncology research in developing countries often needs to be carried out while juggling regular clinical, academic, regulatory, and administrative responsibilities. Young researchers need to find time for research activities and publication. Therefore, for fruitful experimental work and paper writing, researchers must be enthusiastic and invest at least 2 hours a day in thinking about their research problem and paper writing. No research topic is more interesting and important than humans.

Persistently thinking about the problem and summarizing the thoughts in text whenever time permits are essential. If financially viable, a researcher should avail professional language editing services, as this increases the chances of publication.

Until there is sufficient academic and financial support, oncology researchers in developing countries have to come forward with goodwill and passion to learn how to publish and put it into practice; this will help the future researchers attain good research orientation.

## Figures and Tables

**Table 1:** List of journals for publication in medical physics, radiation physics, oncology/radiation oncology, imaging, and biophysics

Journal	SJR (IF, year)	

*Journal of Radiosurgery and SBRT*	0 (0; 2019)	
*Korean Journal of Medical Physics*	0 (0; 2020)	
*Cancer Research, Statistics, and Treatment*	0 (0,2020)	
*Medical Physics International*	0 (0; 2019)	
*Journal of Radiotherapy in Practice*	0.14 (0; 2019)	
*Journal of Medical Imaging and Rad Sciences*	0.202 (0; 2019)	
*Iranian Journal of Medical Physics*	0.233 (0; 2019)	
*Technology and Health Care*	0.247 (0.719; 2019)	
*Indian Journal of Cancer*	0.257 (0.685; 2019)	
*Health and Technology*	0.28 (0; 2019)	
*Journal of Medical Physics and Biophysics*	0.294 (0; 2019)	
*Journal of Medical Physics*	0.294 (0; 2019)	
*Bulletin du Cancer*	0.298 (0.729; 2018)	
*Physical and Engineering Sciences in Medicine*	0.312 (1.006; 2018)	
*Biomedical Physics and Engineering Express*	0.317 (0; 2019)	
*Radiography*	0.358 (0; 2019)	
*Radiation Protection Dosimetry*	0.363 (0.822; 2019)	
*Reports of Practical Oncology and Radiotherapy*	0.412 (0; 2019)	
*Pol Journal of Medical Physics and Engineering*	0.44 (0; 2019)	
*Radiological Physics and Technology*	0.456 (0; 2020)	
*Journal of Cancer Research and Therapeutics*	0.461 (0.842; 2019)	
*Medical Dosimetry*	0.623 (0.886; 2019)	
*Neurochirurgie*	0.437 (0.948; 2018)	
*Stereotactic and Functional Neurosurgery*	0.724 (1.635; 2019)	
*Japanese Journal of Radiology*	0.492 (1.439; 2019)	
*Cancer Radiothérapie*	0.344 (1.014; 2020)	
*Journal of Medical Imaging and Rad Oncology*	0.281 (1.283; 2020)	
*Oncology Research and Treatment*	0.558 (1.967; 2019)	
*La Radiologia Medica*	0.574 (2.0; 2019)	
*Radiation Physics and Chemistry*	0.596 (2.226; 2020)	
*Zeitschrift für Medizinische Physik*	0.605 (2.0; 2019)	
*Asia pacific journal of clinical oncology*	0.611 (2.012; 2018)	
*International Journal of Radiation Biology*	0.618 (2.368; 2019)	
*British Journal of Radiology*	0.766 (2.196; 2019)	
*Brachytherapy*	0.815 (1.853; 2020)	
*Physica Medica*	0.887 (2.458; 2020)	
*Computers in Biology and Medicine*	0.834 (3.434; 2020)	
*Strahlentherapie und Onkologie*	0.909 (2.899; 2019)	
*Clinical and Translational Oncology*	0.833 (2.737; 2019)	
*Radiation Research*	0.972 (2.779; 2019)	
*Computerized Medical Imaging and Graphics*	1.035 (3.750; 2020)	
*Seminars in Radiation Oncology*	1.035 (4.076; 2019)	
*Clinical Oncology*	1.117 (3.113; 2019)	
*Physics in Medicine and Biology*	1.143 (2.883; 2019)	
*Practical Radiation Oncology*	1.178 (2.948; 2019)	
*Acta Oncologica*	1.21 (3.7; 2019)	
*Hematology/Oncology Clinics of North America*	1.232 (3.107; 2019)	
*Medical Physics*	1.275 (3.317; 2019)	
*Current Problem in Cancer*	1.433 (3.264; 2019)	
*The Cancer Journal*	1.491 (2.316; 2019)	
*Radiotherapy and Oncology*	2.003 (4.856; 2019)	
*International Journal of Radiation Oncology Biology Physics*	2.096 (5.859; 2019)	

Journals with article processing charge or other fees		

Journal	SJR (IF, year)	APC

*IOP SciNotes*	0 (0; 2019)	GBP 675
*Physics and Imaging in Rad Oncology*	0 (0; 2019)	EUR 1530
*Technical Innovations and Patient Support in Radiation Oncology*	0 (0; 2019)	EUR 1290
*Clinical and Translational Radiation Oncology*	0 (0; 2019)	EUR 1450
BJR Green	0 (0; 2019)	GBP 2100
BJR|case reports	0 (0; 2019)	GBP 460
*Journal of the Balkan Union of Oncology*	0.392 (1.69; 2019)	EUR 520
*In vivo*	0.458 (1.541; 2019)	USD 800
*Radiation Oncology Journal*	0.829 (0; 2019)	GBP 1790
*Advances in Radiation Oncology*	0.848 (0, 2019)	USD 2000
*Asian Pacific Journal of Cancer Prevention*	0.502 (1.604; 2019)	USD 200
*Technology in Cancer Research and Treatment*	0.526 (2.07; 2019)	USD 2,300
*Journal of Medical Radiation Sciences*	0.608 (1.58; 2019)	USD 2,090
*Journal of Contemporary Brachytherapy*	0.619 (1.62; 2019)	EUR 200
*Medicine* (baltimore)	0.639 (1.55; 2019)	USD 1650
*Journal of Applied Clinical Medical Physics*	0.681 (1.679;2019)	USD 600
*Journal of Radiation Research*	0.715 (1.95; 2019)	GBP 1500
*Radiation Oncology*	1.035 (2.81; 2019)	GBP 1790
*PLOS ONE*	1.023 (2.74; 2019)	USD 1,749
*Physics in Medicine*	1.143 (0; 2019)	USD 1340
*Cancer Research and Treatment*	1.39 (3.76; 2019)	USD 600

Journals that accept case reports		

*Cureus*	eCancer	*Applied Radiation Oncology*

Non-English journals		

*Der Onkologe/the oncologist*	*Japanese Journal of Medical Physics*	*Chinese J of Medical Physics*
*Progress in Medical Physics* (Korea)	*Meditsinskaya Fizika* (Russian)	*Revista de Fisica Medica* (Spain)
*Revista de Bioingenierı’a y Fı’sica Me’dica Cubana* (Cuba)	Revista de Fizica Medicala (Romania)	*Revista Latinoamericana de Fisica* Medica (Spanish)

Journals with low or no SJR (English)		

*International Journal of Radiology and Radiation Therapy*	*Breast Cancer: Current Research*	*Advances in Oncology Research and Treatments*
*International Journal of Cancer Therapy and Oncology*	*Journal of Nuclear Medicine and Radiation Therapy*	*African Journal of Medical Physics Biomedical Engineering and Sciences*
*International Journal of Medical Physics, Clinical Engineering, and Radiation Oncology*	*Insights in Medical Physics*	*Journal of cancer prevention and current research*
*International Medical Physics Journal*	*Journal of medical physics and applied sciences*	*Bangladesh Journal of Medical Physics*

This list exclusive for journal published in the English language. Few journals are multi-linguistic. SJR: Scientific Journal Ranking, IF: Clarivate analytics impact factor, APC: Article processing charge, SBRT: Steriotactic Body radiotherapy, IOP: Institute of Physics, BJR: British Journal of Radiology, GBP: Great Britain Pound, Euro: European currency, USD: United State Dollar

## References

[1] Grech V, Cuschieri S. Write a scientific paper (WASP) – A career-critical skill. Early Hum Dev 2018;117:96–7. 29361328 10.1016/j.earlhumdev.2018.01.001

[2] Maloy S. Guidelines for Writing a Scientific Article. Available from: http://www.sci.sdsu.edu/~smaloy/MicrobialGenetics/topics/scientific-writing.pdf. [Last accessed on 2021 Mar 22].

[3] Aggarwal A, Lewison G, Rodin D, Zietman A, Sullivan R, Lievens Y. Radiation therapy research: A global analysis 2001-2015. Int J Radiat Oncol Biol Phys 2018;101:767–78. 29976487 10.1016/j.ijrobp.2018.03.009

[4] World Bank Country and Lending Groups. Available form: https://datahelpdesk.worldbank.org/knowledgwbase/articles/906519-world-bank-country-and-lending-groups. [Last accessed on 2021 Mar 26].

[5] Gate Research. Available from: https://www.researchgate.net/. [Last accessed on 2021 Mar 26].

[6] Research for Life: Hinari Access to Research for Health Programme. Available from: https://www.who.int/hinari/en/. [Last accessed on 2018 Mar 22].

[7] Bedford JL, Hanson IM, Hansen VN. Comparison of forward- and back-projection in vivo EPID dosimetry for VMAT treatment of the prostate. Phys Med Biol 2018;63:025008. 29165319 10.1088/1361-6560/aa9c60

[8] Clinical Trials: Database of Privately and Publicly Funded Clinical Studies Conducted Around the World. Available from: https://www.clinicaltrials.gov/. [Last accessed on 2018 Mar 22].

[9] Manjali JJ, Gupta T. Critical appraisal of a clinical research paper: What one needs to know. Cancer Res Stat Treat 2020;3:545–51.

[10] Sarkar B, Ray J, Ganesh T, Manikandan A, Munshi A, Rathinamuthu S, Methodology to reduce 6D patient positional shifts into a 3D linear shift and its verification in frameless stereotactic radiotherapy. Phys Med Biol 2018;63:075004. 29480166 10.1088/1361-6560/aab231

[11] Sarkar B, Munshi A, Manikandan A, Roy S, Ganesh T, Mohanti BK, A low gradient junction technique of craniospinal irradiation using volumetric-modulated arc therapy and its advantages over the conventional therapy. Cancer Radiother 2018;22:62–72. 29195796 10.1016/j.canrad.2017.07.047

[12] Science Journal Impact Factor. https://www.scijournal.org/. [Last accessed on 2018 Jun 22].

[13] Scopus List of Journal. Available from: https://www.scopus.com/home.uri. [Last accessed on 2018 Jun 22].

[14] Master Journal List: Science Citation Index. Available form: https://mjl.clarivate.com/home. [Last accessed on 2021 Mar 27].

[15] List of Journals: Cancer Index. Available from: http://www.cancerindex.org/clinks9.htm. [Last accessed on 2021 Apr 09].

[16] Caon M. There are too many medical physics journals! Australas Phys Eng Sci Med 2016;39:813–6. 10.1007/s13246-016-0485-3 27658667

[17] Sarkar B, Wang YX, Cai J. The open access financial model hinders the growth of medical physics research in low- and middle-income countries. Med Phys 2020;47:5972–5. 32367514 10.1002/mp.14220

[18] Zietman AL. Too much impact? Scientific journals and the “impact factor”. Int J Radiat Oncol Biol Phys 2014;90:246–8. 25304783 10.1016/j.ijrobp.2014.07.018

[19] Jalalian M. The story of fake impact factor companies and how we detected them. Electron Physician 2015;7:1069–72. 26120416 10.14661/2015.1069-1072 PMC4477767

[20] The Clarivate Analytics Impact Factor. Available from: https://clarivate.com/webofsciencegroup/essays/impact-factor/. [Last accessed on 2021 Mar 27].

